# 

**Published:** 1879-09

**Authors:** 


| | PAGE. |
|---|---|
| ORIGINAL COMMUNICATIONS: | |
| Antisepsis in Midwifery, by Thomas Lothrop, M. D., | 41 |
| Fracture of the Patella from Muscular Action, by Frank H. Hamilton, M. D., | 55 |
| TRANSLATIONS: | |
| Illumination of the Cavities of the Body by a New Instrument—Nitsche, by A. Osterday, M. D., | 59 |
| SELECTIONS: | |
| Intravenous Injection of Ammonia, | 61 |
| Antiseptic Treatment in Extraction of Cataract, | 65 |
| Transplantation of the Cornea, | 66 |
| Twin Labors, | 67 |
| On the Duration of Life in the Foetus in Utero, after the Mother's Death, | 69 |
| Treatment of Hemorrhoids by Hypodermic Injection of Carbolic Acid, | 70 |
| Battery Fluid, | 71 |
| Diphtheritic Poison, | 71 |
| Treatment of Acne, | 71 |
| Cascara Sagrada in Constipation, | 72 |
| Treatment of Ingrown Toe-Nail, | 72 |
| Jaborandi in the Treatment of Mumps, | 73 |
| Endermic Use of Morphia by "Thimble Blistering," | 73 |
| Poisoning by Sodium Salicylate, | 73 |
| SOCIETY REPORTS: | |
| Buffalo Medical Association, | 74 |
| EDITORIAL: | |
| The Sanitary Condition of Buffalo, | 77 |
| Mortality Table, | 80 |
| Business Notice to Subscribers, | 80 |
| REVIEWS, | 81 |

